# Anthropometric Differences between HIV-Infected Individuals Prior to Antiretroviral Treatment and the General Population from 1998–2007: The AIDS Clinical Trials Group Longitudinal Linked Randomized Trials (ALLRT) Cohort and NHANES

**DOI:** 10.1371/journal.pone.0065306

**Published:** 2013-06-03

**Authors:** Benjamin E. Atkinson, Supriya Krishnan, Gary Cox, Todd Hulgan, Ann C. Collier

**Affiliations:** 1 Madison Clinic, Harborview Medical Center, Seattle, Washington, United States of America; 2 Statistical and Data Analysis Center, Harvard School of Public Health, Boston, Massachusetts, United States of America; 3 Department of Medicine – Infectious Diseases, Duke University Medical Center, Durham, North Carolina, United States of America; 4 School of Medicine, Vanderbilt University, Nashville, Tennessee, United States of America; 5 Division of Allergy and Infectious Diseases, University of Washington School of Medicine, Seattle, Washington, United States of America; Fundacion Huesped, Argentina

## Abstract

**Objective:**

To assess differences in body circumferences and body mass index (BMI, kg/m^2^) between antiretroviral treatment (ART) naïve HIV-infected and HIV-uninfected persons.

**Methods:**

Waist, arm, and thigh circumferences and BMI were measured within the ALLRT and NHANES cohorts between 1998 and 2007. ALLRT is a prospective, longitudinal study of U.S. participants enrolled in randomized HIV treatment studies conducted by the AIDS Clinical Trials Group (ACTG). NHANES is a representative group of the US population. The cohorts were analyzed in two time periods, to account for trends towards increased adiposity. Anthropometrics were displayed in percentiles by age and sex. Multiple linear regression models examined differences between cohorts.

**Results:**

ALLRT had more males (82% versus 48%, p<0.0001), more black participants (32% versus 23%, p<0.0001), and less Hispanics (21% versus 30%, p<0.0001) than NHANES. Mean BMI was smaller in ALLRT males and females compared to NHANES by 1.6–2.4 kg/m^2^ (p<0.0001). Mean waist and arm circumferences in both sexes and time periods were significantly smaller in ALLRT than in NHANES (p<0.0001). Mean thigh circumference in ALLRT was also smaller than NHANES among males (p<0.0001 in both time periods) and females (p = 0.01 in the early time period).

**Conclusions:**

Differences in anthropometrics existed prior to ART initiation, in this large national cohort of HIV-infected individuals, compared to a representative HIV-uninfected cohort, indicating that HIV and its complications have important effects on body shape. Further longitudinal examination of anthropometrics in this HIV-infected cohort may provide additional insight into disease risk.

**Trial Registration:**

NCT00001137 at www.clinicaltrials.gov.

## Introduction

Weight distribution plays an important role in disease risk and mortality. Abdominal obesity, defined as waist circumference (WC) ≥102 cm for men and ≥88 cm for women, increased in the United States between 1999 and 2008 for both sexes [1]. Obesity, or body mass index (BMI) ≥30 kg/m^2^, also increased for both sexes over the same time period. In a 10-year longitudinal study of over 100,000 U.S adults over the age of 50, elevated waist circumference predicted increased all-cause, cancer-related, and cardiovascular mortality [2]. BMI also predicted all-cause, cancer-related, and cardiovascular mortality in a cohort of over 1 million mostly-white volunteers in the U.S., with increased risk in the overweight (BMI 25 to <30 kg/m^2^), obese, and underweight (BMI <18.5 kg/m^2^) subjects compared with healthy weight (BMI 18.5 to <25 kg/m^2^) [3]. Thus, weight distribution and body shape are associated with overall death rates, as well as those from cancer and cardiovascular disease.

Lipodystrophy (peripheral fat loss and/or central fat gain) is a common diagnosis in HIV-infected individuals; and similar to general or abdominal obesity, is an altered distribution of body fat. Between 13% and 62% of HIV-infected persons have been reported to develop lipodystrophy [4]–[6]. Self-report, case diagnosis, and assessment by clinicians are used to diagnose lipodystrophy and its components, although imaging studies are also used as research tools [5]–[7]. Body weight and circumferences correlate well with regional body compositions measured by dual-energy x-ray absorptiometry (DXA) in HIV-infected subjects both naïve to antiretroviral therapy (ART) and during ART [8].

Lipodystrophy negatively affects many aspects of health in people living with HIV. When matched by age, sex, and BMI with HIV-uninfected subjects in the Framingham Offspring Study, Hadigan, et al. reported that male HIV-infected subjects with clinician-diagnosed lipodystrophy had ten-year coronary heart disease (CHD) risk that was significantly higher than controls [9]. In the Fat Redistribution and Metabolic change study (FRAM), elevated WC was positively associated with mortality after 5 years in 922 subjects [10]. Mean depression scores from 250 patients in an urban HIV clinic were significantly higher in those with moderate-to-severe lipoatrophy or with any degree of lipohypertrophy, than subjects without lipodystrophy, after controlling for age, race, sex, drug use, current ART use, and CD4 cell count [11]. In an Italian metabolic clinic, 401 HIV-infected individuals were found to have significant reductions in health-related quality of life with more severe patient-reported lipodystrophy [12]. These data demonstrate that both aspects of lipodystrophy significantly impact the physical and mental health of HIV-infected patients.

Alterations in body weight also have a negative influence on the health of HIV-infected individuals. The Nutrition for Healthy Living (NFHL) cohort, a group of HIV-infected individuals enrolled between 1995–2005 in the Northeast U.S., of whom 66% were taking ART, documented that 18% of its enrollees developed wasting, defined as greater than 10% weight loss [13]. A study of a managed care population reported greater clinic visits and doubling of annual health care costs related to HIV-associated weight loss [14]. Analyses of a HIV-infected military cohort, between 1996 and 2008, emphasized the importance of a healthy weight or overweight status, adjusted for time on ART. These ranges were associated with greater mean increases in CD4 lymphocyte counts, compared to obese or underweight subjects [15]. In ART-naïve patients at an outpatient clinic in Nashville, Tennessee, CD4 lymphocyte count increased most after 12 months of ART in those with a baseline BMI between 25–30 kg/m^2^ [16]. Thus, normal or overweight BMI appears to have positive benefits in reduced health care costs and greater immune reconstitution in HIV-infected individuals.

The aim of this study was to compare anthropometric measurements during similar time periods in a large group of HIV-infected, ART-naïve adults of both sexes to HIV-uninfected men and women. We hypothesized that HIV-infected males and females would have smaller body circumferences and BMI than the HIV-uninfected persons.

## Methods

### Ethics Statement

Subjects (or their parent or legal guardian if they were a minor) provided written informed consent and institutional review board (IRB) approval for ALLRT (AIDS Clinical Trials Group (ACTG) Longitudinal Linked Randomized Trials) was obtained by each site.

### Study Population

#### HIV-Infected individuals

ALLRT is a prospective, longitudinal, observational cohort of U.S. adults enrolled in randomized HIV treatment studies (parent studies) conducted by the ACTG. The study design and rationale of ALLRT have been previously described [17]. After completion of their parent study, participants in ALLRT continue in long-term follow-up. This study utilized data from six ALLRT parent studies that enrolled participants who were ARV-naïve [18]–[23].

#### HIV-Uninfected individuals

We used data from the National Health and Nutrition Examination Survey (NHANES) from 1999 to 2006. NHANES is a national survey of the U.S. population conducted annually which focuses on health and nutritional status, using interviews and physical exams. Additional details about NHANES can be found elsewhere [24]. HIV antibody test results were available in NHANES for approximately 60% of subjects aged 18–49 years who didn’t refuse the test; only 0.6% were positive. Given the small proportion of HIV-infected participants, these subjects were not excluded from our analyses of NHANES data. Only data from NHANES subjects aged 17–74 years were included to match the age range of participants in ALLRT. NHANES race/ethnicity definitions of ‘Mexican-American’ and ‘Other Hispanics’ were collapsed into a category of ‘Hispanic’ to correspond to the definitions used in ALLRT.

### Data Collection

#### HIV-Infected individuals

Data collection for ALLRT at each visit (every 16 weeks) includes medical and medication history, weight, and vital signs. Height is measured at parent trial entry. Measurements of waist circumference (WC), arm circumference (AC) and thigh circumference (TC) were initially measured at parent trial entry (baseline) prior to ART initiation, and then every 48 weeks thereafter. For some individuals, the first available anthropometric measurement was 16 weeks after baseline due to the timing of their ALLRT entry visit. For these individuals, the anthropometric measure at week 16 was considered as baseline.

Anthropometrics among ALLRT participants outside of pre-determined extreme ranges were not included (body weights had to be between 23–182 kg, WC between 61–160 cm, AC between 18–51 cm, and height between 120–213 cm).

#### HIV-Uninfected individuals

NHANES participants completed an interview and standardized medical exams. Anthropometric evaluations included height, weight, WC, AC, and TC [24]. NHANES data are available continuously from 1999 to the present and datasets in 2-year intervals are available to the public on the Centers for Disease Control (CDC) website [25]. We used data from the NHANES years closest to the enrollment years for the ALLRT cohort. To account for increases in body weight and WC in the U.S. population over time [1], we divided the NHANES and ALLRT subjects into two groups: those enrolled in 1999–2002 and 2003–2006 for NHANES, and 1998–2002 and 2003–2007 for ALLRT. NHANES checks for measurement and recording errors at the 1^st^ and 99^th^ percentiles of anthropometric data, based on NHANES III reference values, and excludes data from its datasets thought to be erroneous [26]. Ten percent of NHANES participants with any missing anthropometric measures were excluded.

### Data Analysis

Categorical variables were compared between ALLRT and NHANES using chi-square tests. Continuous variables were compared using the Wilcoxon test. Summaries of BMI and anthropometric measures by sex and age were presented using percentiles. The prevalence of BMI ≤20 kg/m^2^ was reported, as this cut-off predicts increased mortality in HIV-uninfected populations [3], and has been used to indicate wasting in HIV-infected populations [27]. To compare anthropometric measures between ALLRT and NHANES subjects of the same sex, equality of means was tested using linear regression, adjusting for age, race/ethnicity, square root of height (√height ), diabetes history and smoking history. The inclusion of √height in regression analyses accounts for body circumference differences noted between short and tall adults within ethnicities and age groups, as noted in a recent analysis of NHANES III [28]. Age and race-adjusted WC and hip circumference measures, in this representative population, scaled to height with powers between 0.45 and 0.8 in men and women, for an average of approximately 0.5–much different than the height-squared factor included in the calculation of BMI. For all analyses, p<0.05 was considered statistically significant.

## Results

### Demographics of Study Population

**Figure 1:**
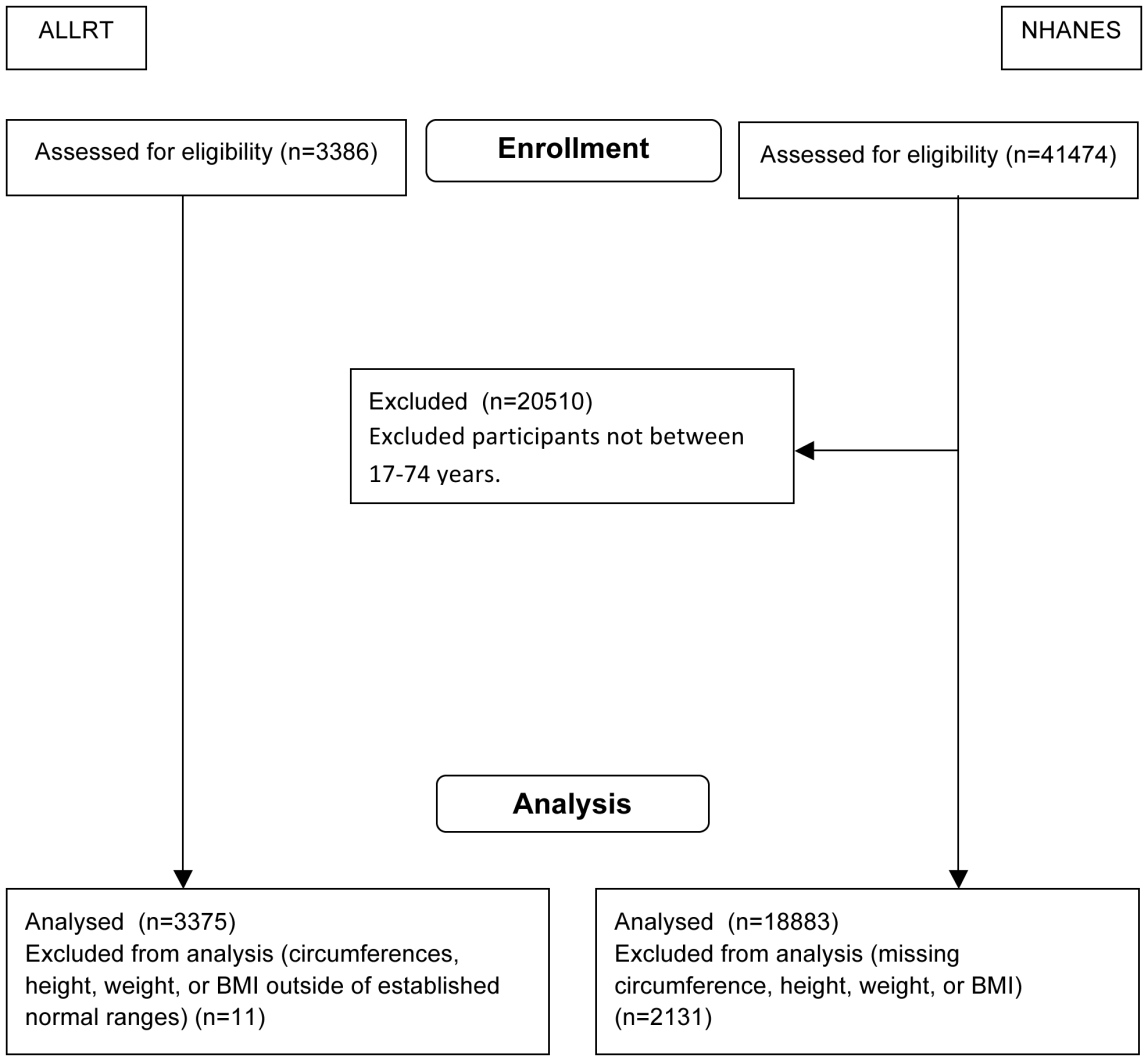
Flow diagram. NHANES: National Health and Nutrition Examination Survey ALLRT: AIDS Clinical Trials Group Longitudinal Linked Randomized Trials.

A total of 3375 ALLRT and 18,833 NHANES participants were included in this analysis, as seen in Figure 1. Demographic characteristics of the ALLRT and NHANES participants during each of the two periods are presented in Table 1. ALLRT subjects were more likely to be male (82% and 48% in ALLRT and NHANES, respectively, p<0.0001). ALLRT enrolled a higher proportion of black subjects and a smaller proportion of Hispanics than NHANES in both periods (p<0.0001).

**Table 1:** Baseline characteristics of the study population.

Baseline Characteristics	ALLRT 1998–2002 (N = 1611)	NHANES 1999–2002 (N = 9483)	ALLRT 2003–2007 (N = 1764)	NHANES 2003–2006 (N = 9350)
	*N (%)*	*N (%)*	*N (%)*	*N (%)*
Sex, male	1307 (81)	4550 (48)^a^	1461 (83)	4494 (48)^a^
Race/Ethnicity				
White, non-Hispanic	731 (45)	3991 (42)^a^	749 (43)	4182 (45)^a^
Black, non-Hispanic	500 (31)	2002 (21)^a^	570 (32)	2301 (25)^a^
Hispanic	340 (21)	3139 (33)^a^	385 (22)	2457 (26)^a^
Other	40 (3)	351 (4)^a^	60 (3)	410 (4)^a^
Cigarette smoking history				
Ever smokers	956 (60)	4817 (51)^a^	966 (56)	4643 (50)^a^
Never smokers	646 (40)	4545 (48)^a^	759 (44)	4594 (50)^a^
Diabetes diagnosis				
Yes	48 (3)	758 (8)^a^	60 (3)	828 (9)^a^
No	1563 (97)	8721 (92)^a^	1704 (97)	8516 (91)^a^
	*Median (Q1, Q3)*	*Median (Q1, Q3)*	*Median (Q1, Q3)*	*Median (Q1, Q3)*
Age, years	37 (31, 43)	39 (24, 55)^b^	38 (31, 45)	38 (24, 54)^c^
Height, cm	174 (168, 180)	167 (160, 175)^a^	174 (168, 180)	168 (161, 175)^a^
Weight, kg	74 (65, 84)	76 (64, 89)^a^	75 (66, 86)	77 (65, 91)^a^
CD4+ T-cells/mm^3^	215 (67, 369.5)	–	217 (74, 325)	–
HIV-1 RNA, log_10_copies/mL	4.9 (4.4, 5.5)	–	4.8 (4.4, 5.1)	–

a NHANES significantly different from ALLRT (p<0.0001) for the corresponding time period.

b NHANES significantly different from ALLRT (p<0.05) for the corresponding time period.

c NHANES not significantly different from ALLRT (p>0.1) for the corresponding time period.

In ALLRT, during the first time period, females and males were of similar age, but during the second time period, women were slightly older (median age in years (Q1, Q3) = 38 (31, 45) in males, 40 (33, 46) in females, Wilcoxon test p-value = 0.01). In NHANES, males were slightly older than females in each time period (median age in years (Q1, Q3) = 40 (23,56) in males, 37 (24, 54) in females, Wilcoxon test p-value = 0.02 in the first time period) (median age in years (Q1, Q3) = 39 (24, 55) in males and 36 (24, 53) in females, Wilcoxon test p-value = 0.004 in the second time period). Rates of smoking were significantly higher among ALLRT participants, but prevalence of diabetes mellitus was significantly lower than in NHANES (Wilcoxon test p-value<0.0001 in each time period).

### Unadjusted Anthropometric Data for HIV-infected Individuals

The 25^th^, 50^th^, and 75^th^ percentiles of unadjusted BMI in ALLRT are presented in Table 2. The median BMI of younger males (20–29 and 30–39 years) in both time periods, as well as older males (40–49 and 50–59 years) in the earlier time period, was in the healthy BMI range. The median BMI in ALLRT for females was in the overweight range, with the exception of the youngest age group in the later time interval that had median BMI of 24 kg/m^2^. Within each time period, females had larger BMI than males (p<0.0001); the differences in medians within age groups ranged from 0.5–3 kg/m^2^.

**Table 2:** BMI (kg/m^2^), by sex and age in ALLRT.^a^ (Unadjusted percentiles).

	1998–2002				2003–2007			
	N	Percentile			N	Percentile		
Age, years		25^th^	50^th^	75^th^		25^th^	50^th^	75^th^
*Male*								
20–29	220	21.5	23.5	26.0	282	21.0	23.0	25.5
30–39	575	22.0	24.5	27.0	528	22.5	24.5	27.5
40–49	369	22.0	24.5	27.0	441	23.0	25.0	28.0
50–59	104	22.0	23.5	26.7	159	23.0	25.0	28.5
*Female*								
20–29	48	23.2	26.0	30.5	43	21.5	24.0	28.0
30–39	121	22.5	27.0	32.0	106	23.5	27.5	33.5
40–49	87	21.5	25.0	29.0	107	22.5	26.5	32.0
50–59	32	22.2	25.7	29.7	40	22.7	26.2	31.0

a ALLRT participants <20 and >59 years were excluded due to limited sample size.

Overall, 4%, 51%, 32%, and 14% of ALLRT participants were in the underweight, healthy, overweight, and obese BMI categories, respectively. Two percent, 34%, 33%, and 31% of NHANES participants fell into these categories. Within the entire ALLRT cohort, 9.4% of participants had a BMI less than 20 kg/m^2^. These rates were 9.7% to 9.2% in the earlier and later time periods, respectively. In the NHANES cohort, the overall prevalence of BMI less than 20 kg/m^2^ was 6.7%; 7% in the first time period, and 6.4% in the second time period.

As shown in Table 3, females in ALLRT had larger median WC than men in ALLRT in the two younger age ranges (20–29 and 30–39 years); however the differences were not statistically significant. For both men and women, median WC were larger (p<0.05) in the two older age groups (40–49 and 50–59 years) during the later time period compared to the earlier by 1.75 to 5 cm for women and 1.5 cm for men.

**Table 3:** Waist circumference (cm), by sex and age in ALLRT.^a^ (Unadjusted percentiles).

	1998–2002				2003–2007			
	N	Percentile			N	Percentile		
Age, years		25^th^	50^th^	75^th^		25^th^	50^th^	75^th^
*Male*								
20–29	144	78.0	83.5	88.7	269	77.0	83.0	91.5
30–39	374	80.5	87.0	94.0	504	82.0	87.5	96.0
40–49	248	82.5	88.5	95.0	422	84.0	90.0	97.0
50–59	66	82.0	90.0	96.5	149	85.0	91.5	101.5
*Female*								
20–29	30	78.0	86.0	98.0	42	75.5	83.5	90.0
30–39	77	78.0	90.0	102.0	102	81.0	89.8	104.0
40–49	61	77.5	84.5	94.5	104	82.2	89.5	99.0
50–59	20	82.7	89.0	94.5	38	83.0	90.8	100.5

a ALLRT participants <20 and >59 years were excluded due to limited sample size.

Overall median AC (Table 4) was smaller in women than men in ALLRT (p<0.05) within each time period; the differences in medians by age groups ranged from 0.8 to 2 cm, with the exception of no difference in youngest age range in the early time period. For TC (Table 5), women in ALLRT had larger median circumferences (p<0.0001) than men in ALLRT in the early time period; the differences in medians by age groups ranged from 1.7–5 cm. For the second time period, median thigh circumferences were higher among females than males (not statistically significant) for all age groups with the exception of females aged 40–49 years.

**Table 4:** Arm circumference (cm), by sex and age in ALLRT.^a^ (Unadjusted percentiles).

	1998–2002					2003–2007			
	N	Percentile				N	Percentile		
Age, years		25^th^	50^th^	75^th^			25^th^	50^th^	75^th^
*Male*									
20–29	142	27.5	29.5		32.0	238	27.5	29.5	32.0
30–39	369	28.5	31.0		33.5	404	28.5	31.0	33.5
40–49	244	28.0	31.0		33.0	343	29.0	31.5	33.5
50–59	67	28.0	30.0		32.5	129	28.0	30.5	33.0
*Female*									
20–29	30	26.0	29.5		35.5	30	26.0	28.0	31.0
30–39	78	26.5	30.7		35.0	82	27.5	30.2	35.0
40–49	60	27.0	29.0		31.5	80	26.5	30.5	33.7
50–59	19	26.0	28.0		33.5	29	28.5	29.5	32.5

a ALLRT participants <20 and >59 years were excluded due to limited sample size.

**Table 5:** Thigh circumference (cm), by sex and age in ALLRT.^a^ (Unadjusted percentiles).

	1998–2002				2003–2007			
	N	Percentile			N	Percentile		
Age, years		25^th^	50^th^	75^th^		25^th^	50^th^	75^th^
*Male*								
20–29	147	46.0	50.0	54.0	72	45.5	50.2	54.5
30–39	412	47.0	51.0	55.0	128	48.5	52.0	56.0
40–49	270	46.0	50.0	53.5	108	48.5	51.5	55.0
50–59	81	44.0	48.0	51.5	32	46.0	51.0	56.7
*Female*								
20–29	31	47.5	55.0	60.0	12	49.7	51.2	60.7
30–39	77	49.5	54.0	59.5	20	49.0	59.2	64.0
40–49	64	46.0	51.7	57.2	26	44.0	49.5	55.0
50–59	27	45.0	50.0	56.0	10	43.5	52.7	60.0

a ALLRT participants <20 and >59 years were excluded due to limited sample size.

### Adjusted Anthropometric Data comparing HIV-infected to HIV-uninfected Individuals

**Figure 2:**
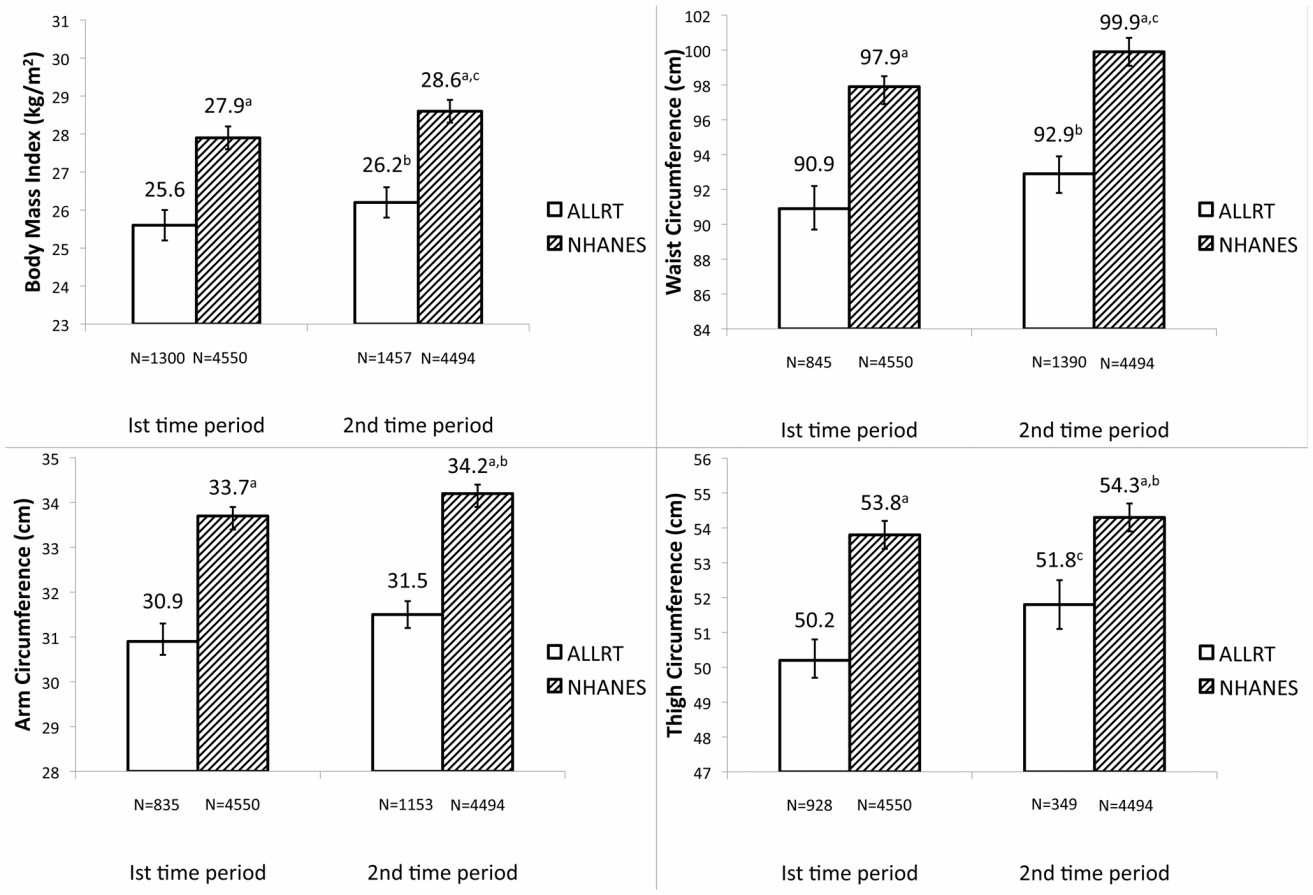
Mean (95% CI) anthropometrics among males, adjusted* for age, race, √height, smoking history and diabetes history. NHANES: National Health and Nutrition Examination Survey ALLRT: AIDS Clinical Trials Group Longitudinal Linked Randomized Trials *Age centered at 40 years; reference for race is white; reference for smoking history is non-smoker; reference for diabetes history is no diabetes history. BMI not adjusted for √height. ^a^ NHANES significantly different from ALLRT (p<0.0001). ^b^ Significantly different from 1^st^ time period (p≤0.05). ^c^ Significantly different from 1^st^ time period (p≤0.0001).

**Figure 3:**
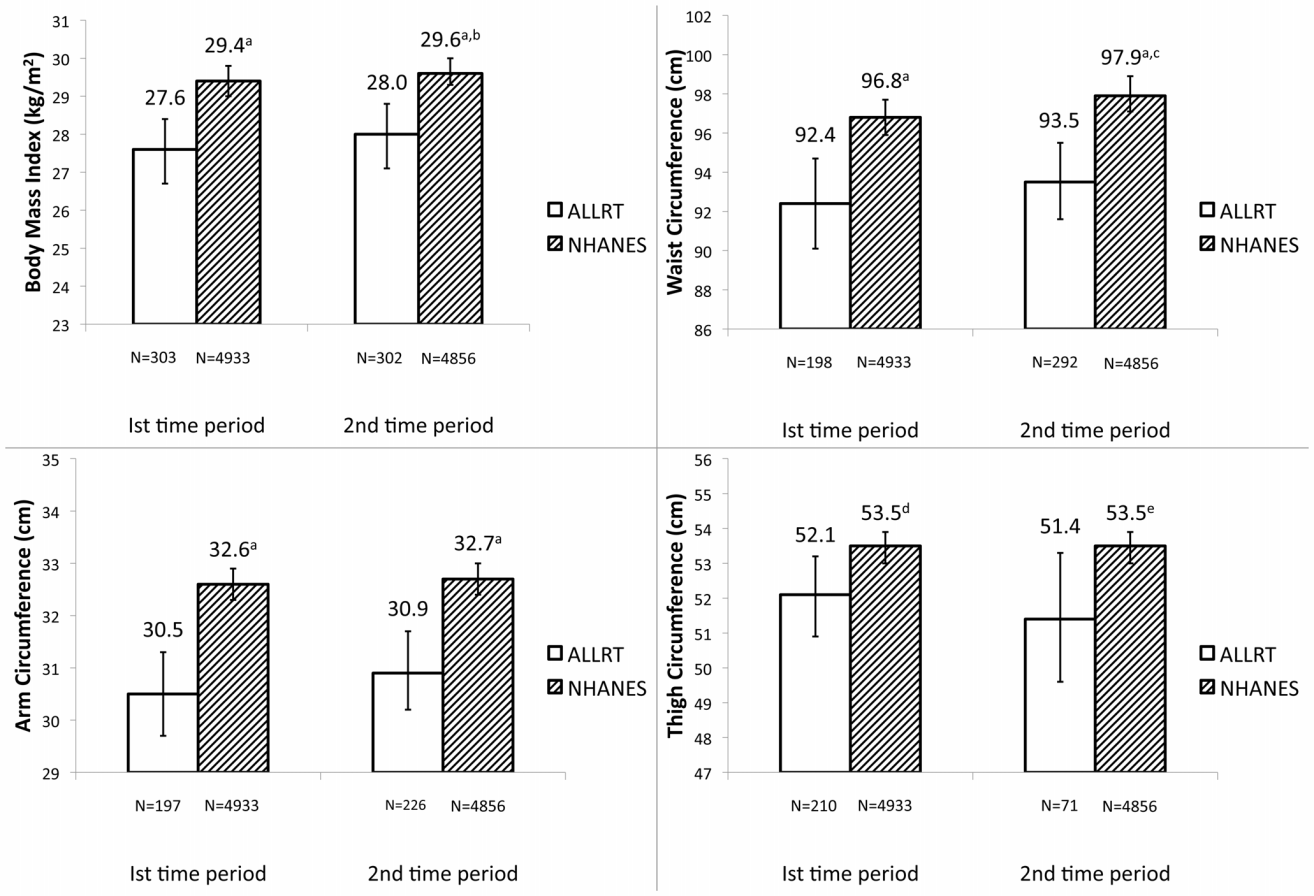
Mean (95% CI) anthropometrics among females, adjusted* for age, race, √height, smoking history and diabetes history. NHANES: National Health and Nutrition Examination Survey ALLRT: AIDS Clinical Trials Group Longitudinal Linked Randomized Trials *Age centered at 40 years; reference for race is white; reference for smoking history is non-smoker; reference for diabetes history is no diabetes history. BMI not adjusted for √height. ^a^ NHANES significantly different from ALLRT (p<0.0001). ^b^ Significantly different from 1^st^ time period (p = 0.02). ^c^ Significantly different from 1^st^ time period (p<0.0001). ^d^ NHANES significantly different from ALLRT (p = 0.01). ^e^ NHANES significantly different from ALLRT (p = 0.03).

Figure 2 (males) and Figure 3 (females) show the comparison of mean BMI, WC, AC, and TC, for each time period in ALLRT and NHANES, adjusted for age, race/ethnicity, √height, diabetes history and smoking history. Mean BMI was 2.3 kg/m^2^ and 2.4 kg/m^2^ smaller (both p<0.0001) in ALLRT compared to NHANES in males in the early and later time periods, respectively. Females in ALLRT also had significantly smaller mean BMI than NHANES in the early and later time periods, by 1.8 kg/m^2^ and 1.6 kg/m^2^ (both p<0.0001), respectively. Compared to the first time period, adjusted mean BMI was slightly higher in ALLRT in the second time period (p = 0.05 for males). Similarly, adjusted mean BMI was higher in the second time period in NHANES (p≤0.0001 in males, p = 0.02 in females).

Mean WC was smaller for both sexes in both time periods in ALLRT compared with NHANES. For both time periods, males in ALLRT had a 7 cm smaller mean WC than NHANES, and females had a 4.4 cm smaller mean WC (all p<0.0001). Higher mean WC in the second time period compared to the first were seen among males in ALLRT (p≤0.05) and both sexes in NHANES (both p≤0.0001).

Mean AC and TC showed the same relationship among both sexes–significantly smaller circumferences in ALLRT than in NHANES at each time period. Mean AC was not different between time periods within ALLRT, but was significantly larger in the second time period in NHANES (p≤0.05). The difference in TC in females between ALLRT and NHANES was smaller than in men. For example, in the first time period, males in ALLRT had TC 3.6 cm smaller than NHANES, and females in ALLRT were 1.4 cm smaller than NHANES. There was no difference in the mean TC between time periods within females in ALLRT, and both sexes in NHANES; however the mean TC among males in ALLRT was higher in the second time period compared to the first period (p = 0.001).

Adjustment for BMI instead of √height (Table 6) decreased the differences in circumferences for waist, arm, and thigh in ALLRT in both time periods, compared with NHANES. With the adjustment for BMI, WC was more similar in ALLRT and NHANES among females in the first time period (p = 0.9), but still larger in NHANES in the later time period only (p = 0.03). Using BMI to adjust the data comparing females suggested larger TC in ALLRT than in NHANES in both time periods with this model in contrast to the results with √height used to adjust the data.

**Table 6:** Mean (95% CI) anthropometrics before ART, adjusted* for age, race, BMI, smoking history and diabetes history.

	ALLRT 1998–2002 (N = 1307)		NHANES 1999–2002 (N = 4550)			ALLRT 2003–2007 (N = 1461)		NHANES 2003–2006 (N = 4494)		
	N	Mean (95% CI)	N	Mean (95% CI)	p-value	N	Mean (95% CI)	N	Mean (95% CI)	p-value
*Males*										
BMI (kg/m^2^)	1300	25.6 (25.2, 26.0)	4550	27.9 (27.6, 28.2)	<.0001	1457	26.2 (25.8, 26.6)	4494	28.6 (28.3, 28.9)	<.0001
Waist Circumference (cm)	845	93.5 (93.0, 93.9)	4550	94.9 (94.6, 95.2)	<.0001	1390	94.8 (94.4, 95.2)	4494	96.1 (95.8, 96.4)	<.0001
Arm Circumference (cm)	835	31.7 (31.5, 31.9)	4550	33.0 (32.8, 33.0)	<.0001	1153	32.2 (32.0, 32.3)	4494	33.1 (33.0, 33.3)	<.0001
Thigh Circumference (cm)	928	51.3 (51.1, 52.6)	4550	52.6 (52.4, 52.7)	<.0001	349	52.8 (52.9, 53.1)	4494	53.1 (52.9, 53.3)	0.02
*Females*										
BMI (kg/m^2^)	303	27.6 (26.7, 28.4)	4933	29.4 (29.0, 29.8)	<.0001	302	28.0 (27.1, 28.8)	4856	29.6 (29.3, 30.0)	<.0001
Waist Circumference (cm)	198	94.1 (93.1, 95.2)	4933	94.2 (93.8, 94.6)	0.9	292	94.4 (93.5, 95.3)	4856	95.3 (94.9, 95.7)	0.03
Arm Circumference (cm)	197	31.2 (30.8, 31.4)	4933	31.7 (31.6, 31.8)	0.0001	226	31.2 (30.9, 31.5)	4856	31.8 (31.7, 31.9)	<.0001
Thigh Circumference (cm)	210	53.0 (52.5, 53.5)	4933	52.2 (51.9, 52.4)	0.001	71	52.6 (51.7, 53.4)	4856	52.2 (51.9, 52.4)	0.3

* Age centered at 40 years and BMI at 27 kg/m^2^; reference for race is white; reference for smoking history is non-smoker; reference for diabetes history is no diabetes history.

## Discussion

To our knowledge, this is the largest study comparing the anthropometrics of HIV-infected individuals to the general U.S. population. An important, but not unexpected finding is that most anthropometric measures were significantly smaller in this HIV-infected ALLRT cohort, with advanced HIV disease, prior to the initiation of ART versus the HIV-uninfected population in NHANES. Given the temporal increases noted in all anthropometrics, with the exception of TC among females, our use of two separate time periods is justifiable.

The significance of BMI, body shape changes, and wasting in HIV care is evolving. Between 1996–2006, the percentage of deaths as a result of HIV and wasting/cachexia, fell from 8.5% to 4.9% of all HIV-related deaths in the US, based on death certificates [29]. Earlier treatment may contribute to less HIV-associated wasting, since all HIV-infected are recommended to start treatment [30]. Between 1995–2005, the NFHL cohort documented wasting using 3 definitions, one of which was BMI <20 kg/m^2^ [27]. These authors noted that 8% of 466 subjects with 6 months of follow-up had wasting by their BMI criteria. This is similar to our results, in which 9.4% of HIV-infected subjects had a BMI <20 kg/m^2^. Though wasting may be decreasing overall in untreated persons with HIV in the US, when defined by low BMI it may reflect the national trend of increasing body weight in the US population. Fewer ALLRT and NHANES participants were below a BMI of 20 kg/m^2^ in the later time period.

While less HIV-infected individuals may be affected by wasting, extremely high body weight may adversely affect health. Crum-Cianflone, et al. analyzed a large US military HIV cohort and noted that after starting ART, obese and underweight subjects had smaller gains in CD4 T-cell count relative to subjects with normal or overweight BMI [15]. HIV-infected patients in the obese BMI range at an outpatient clinic in Tennessee also had less CD4 T-cell count gain over the first 12 months of ART, compared to overweight patients (p = 0.03) [16]. HIV-infected subjects starting ART may especially be at a health disadvantage if underweight, or obese.

Our results indicate that WC prior to ART is smaller in both men and women when compared to a HIV-uninfected cohort. Waist circumferences have been measured in some other studies involving HIV-infected subjects naïve to ART. Visnegarwala, et al. documented a smaller mean WC between 1999 and 2002, in a cohort (n = 422) with more advanced HIV disease and lower CD4 lymphocyte counts than ALLRT [31]. Mean WC was reported by race and sex, and ranged from 83.8–88.4 cm for males and 78.7–88.9 cm for females, which are below our mean values of 90.9 cm for males and 92.4 cm for females in the 1998–2002 ALLRT time period. The percentage of non-white subjects in this study (66.4–96.7% non-white versus 55–57% in ALLRT) may contribute to differences in the results, since Non-Hispanic Black and Mexican-American males generally have smaller WC [32]. In the general U.S. population, large WC is reported to be a predictor of mortality, as was seen in the Cancer Prevention Study II Nutrition Cohort [2], and the NIH-AARP study [33]. In HIV-infected individuals, a relationship between large WC and mortality has also been reported. After 5 years of follow-up in the FRAM study, increased WC at baseline was significantly associated with mortality in multivariate models. However, these subjects were largely taking ART (over 80% reported current use) and had higher CD4 cell counts (333–419 cells/mm^3^) than our cohort [10]. In a cross-sectional analysis of 666 HIV-infected and 242 HIV-uninfected individuals in the FRAM study, larger WC was found to correlate significantly with insulin resistance and HDL-cholesterol [34]. Waist circumferences in untreated HIV infection, while still smaller than the general US population, appear to carry similar risk for mortality and metabolic disturbances.

Arm circumference, as a fat-free mass indicator, may also be an important indicator of mortality. In both the NHANES I and II longitudinal studies [35], and the European cohorts of the Seven Countries Study [36], larger AC predicted lower all-cause mortality after 14.6, 12.9, and 40 years, respectively. Additionally, the FRAM study recently analyzed 5-year follow-up anthropometric data in their HIV-infected cohort, over 80% of which were taking ART. Their results concluded that lower mid-arm muscle circumference at baseline predicted higher mortality in a multivariate model that included traditional CVD and HIV-related factors [10]. The smaller AC found in our untreated HIV-infected cohort suggest these individuals may have a greater risk of death.

Thigh circumferences in females with untreated HIV aren’t widely described in the literature; however reports describe smaller values than the HIV-uninfected subjects in our study. In the Women’s Interagency HIV Study (WIHS), 734 HIV-infected women on stavudine had a mean TC 4 cm smaller compared to 698 HIV-uninfected controls (p<0.01) at baseline visits between 1999–2005 [37]. Analysis of the Multicenter AIDS Cohort Study (MACS) showed that ART naive HIV-infected males had a 2 cm smaller mean TC than HIV-uninfected controls in 1999 [38]. Smaller TC may be associated with a higher risk of CVD and total mortality, as seen in the MONICA prospective study of 2816 HIV-uninfected adults in Denmark [39]. They reported increased risk of mortality after 12.5 years of follow-up in persons below the median TC (55 cm for males and 55.5 cm for females). Similarly for HIV-infected persons, the FRAM study has documented increased CVD risk for low leg subcutaneous fat, as well as increased 5-year mortality for decreased leg muscle mass [10], [40]. Thus, the smaller TC in our HIV-infected subjects in ALLRT may be associated with higher risk for poor health outcomes.

Our study has limitations, notably the inability to discern between different types of tissue in the areas measured. However, a sub-study of one trial included in the present study, ACTG 384, noted significant correlation between body circumferences and DXA measurements prior to, as well as 64 weeks after starting ART [8]. Additionally, the Abacavir versus d4T Plus Efavirenz (ABCDE) study in Spain determined that the percent of limb fat loss required to become clinically evident after ART initiation, based on visual assessment, was 30 percent [41]. Therefore, while the change in circumferences correlate to those seen through imaging, circumference changes likely are not as sensitive a metric and represent large changes in body tissue volume. Information on health habits other than smoking, such as diet and exercise is also unavailable in the ALLRT study. While improvements in exercise will affect anthropometrics positively in HIV-infected persons [42], dietary alterations may have little effect [43], though small studies have found benefits from adherence to a Mediterranean diet [44], [45]. Last, ALLRT was composed of research study participants, which may affect the generalizability of our findings. However, participation in ALLRT may minimize the differences from the general U.S. population, since participants may have had better overall health than other HIV-infected persons.

In summary, these data demonstrate that differences in anthropometrics between HIV-infected subjects in ALLRT and HIV-uninfected subjects in NHANES exist prior to ART and are statistically and clinically significant. Studies using DXA and MRI have demonstrated associations between tissue distribution and HIV disease. Anthropometrics are a simpler, more clinically feasible method to quantify body changes that can be easily visualized by both patient and medical care providers. Additional studies are needed in this HIV-infected cohort to examine the associations between anthropometrics and future disease risk in HIV infection.
